# Control Principles of Neural Dynamics Revealed by the Neurobiology of Timing

**DOI:** 10.1146/annurev-neuro-091724-015512

**Published:** 2025-01-29

**Authors:** Gabriel M. Stine, Mehrdad Jazayeri

**Affiliations:** 1 Department of Brain and Cognitive Sciences, McGovern Institute for Brain Research, Massachusetts Institute of Technology, Cambridge, Massachusetts, USA;; 2 Howard Hughes Medical Institute, Massachusetts Institute of Technology, Cambridge, Massachusetts, USA

**Keywords:** timing, neural computation, neural population dynamics, flexible behavior

## Abstract

Cognition unfolds dynamically over flexible timescales. A major goal of the field is to understand the computational and neurobiological principles that enable this flexibility. Here, we argue that the neurobiology of timing provides a platform for tackling these questions. We begin with an overview of proposed coding schemes for the representation of elapsed time, highlighting their computational properties. We then leverage the one-dimensional and unidirectional nature of time to highlight common principles across these coding schemes. These principles facilitate a precise formulation of questions related to the flexible control, variability, and calibration of neural dynamics. We review recent work that demonstrates how dynamical systems analysis of thalamocortical population activity in timing tasks has provided fundamental insights into how the brain calibrates and flexibly controls neural dynamics. We conclude with speculations about the architectural biases and neural substrates that support the control and calibration of neural dynamics more generally.

## INTRODUCTION

Rushing through a crowded, urban sidewalk, we effortlessly time our stops, lateral movements, and speed to avoid collisions. We are constantly predicting when things will occur and using this information to decide when to act. This is reason enough to understand the cellular, circuit, and computational mechanisms that underlie timing behavior. Yet, our sense of time possesses unique properties that render it particularly suitable for studying the relationship between the brain and behavior in dynamic environments more generally.

First, time intervals are conveniently one-dimensional. Contrast this to, say, vision, where we need tens or hundreds of variables to describe a unique scene or face (Chang & Tsao 2017, Meytlis & Sirovich 2007, Olivia et al. 2004). Consequently, if we want to understand how the brain learns time intervals, flexibly anticipates events, and produces well-timed movements, we need to track down a single well-defined latent variable in the brain. Doing the same in vision or any other rich sensory system will be far more challenging. Therefore, timing offers a convenient model system for exploring broader principles of neural computation and cognition.

Second, unlike other senses, our perception of time does not involve sensory transduction from the external world. Instead, it relies on internally generated dynamics—representations of time are inherently latent. Thus, studying these representations will help uncover general principles of the organization and dynamic representation of latent variables, which are a crucial building block of all cognitive processes.

This article does not provide a comprehensive review of the timing literature—many excellent reviews already exist (Addyman et al. 2016; Buhusi & Meck 2005; Mauk & Buonomano 2004; Merchant & de Lafuente 2014, 2024; Merchant et al. 2013a; Muller & Nobre 2014; Paton & Buonomano 2018; Tsao et al. 2022). We begin by developing a classification of proposed coding schemes for the representation of time within the millisecond to seconds time range across models, brain areas, species, and tasks, exposing their underlying similarities and differences. We then use a state space framework to highlight the common computational properties of these coding schemes as different embeddings of a one-dimensional latent variable in neuronal population activity. Finally, we focus on recent work on the neurobiology of a battery of simple time interval production tasks that have afforded general insights about calibration and flexible control of neural dynamics.

## ENCODING OF TIME BY NEURONAL POPULATIONS

Time is one-dimensional, and unidirectional. Here, we provide a general classification of various neural coding schemes that respect these properties and are observed in animals performing interval timing tasks.

### Ramping Activity

**Figure 1:**
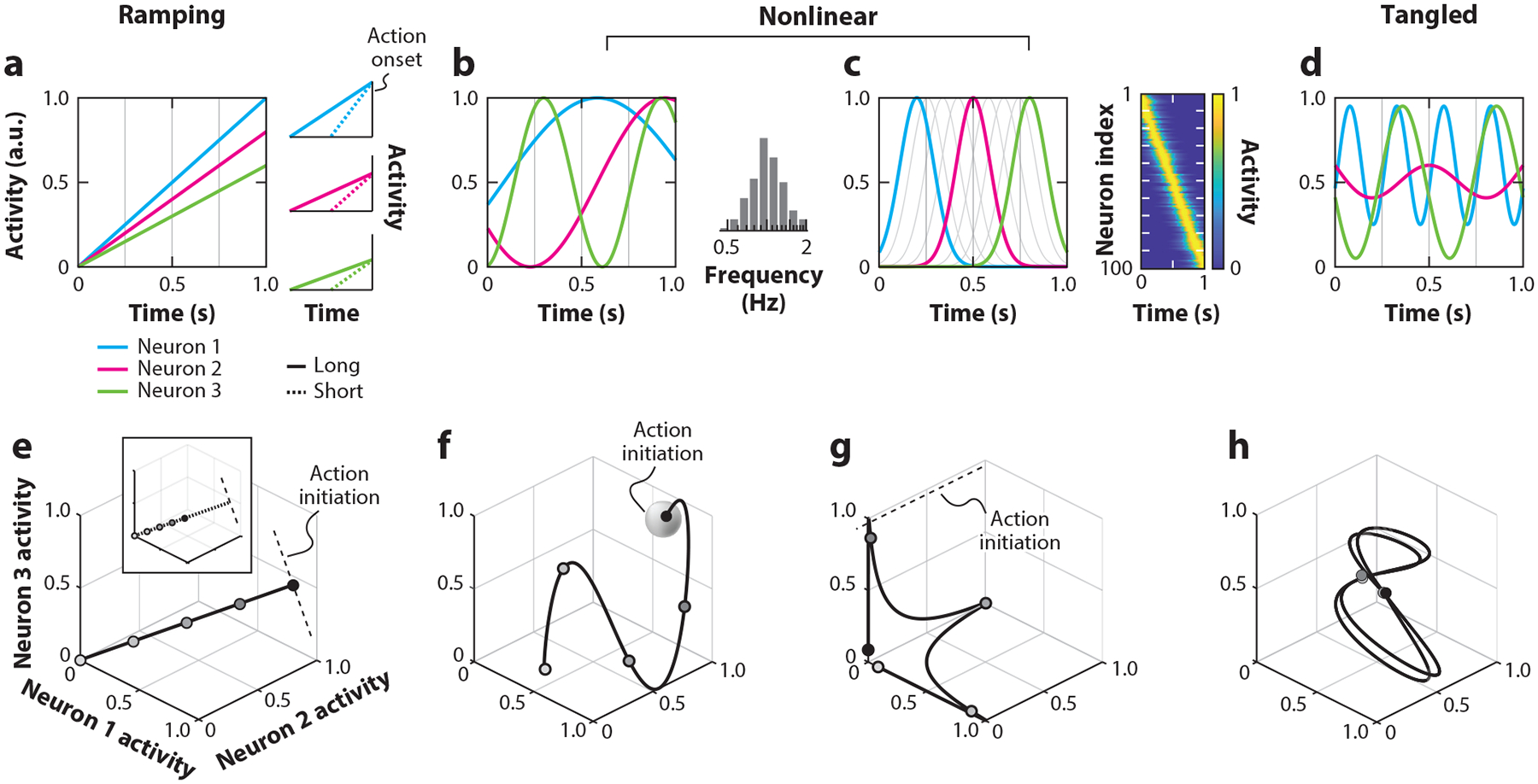
Illustration of different coding schemes for interval timing. (*a*) Ramping scheme for three example neurons. The activity increases linearly with time up to a maximum at the time of action initiation. The only difference between the neurons is the terminal activity level. The three plots on the right show activity profiles of the same three neurons aligned to the time of action initiation during the production of either a long (*solid line*) or short (*dashed line*) interval. (*b*) Nonlinear scheme for three example neurons. The activity of each neuron has a unique nonlinear relationship with elapsed time. In this example, the activity profiles are modulated sinusoidally with different frequencies. The histogram on the right shows a schematic of a population of neurons with sinusoidal activity modulated across a diverse set of frequencies. (*c*) Nonlinear scheme instantiated by sequential activity. In this scheme, each neuron is tuned to a specific point along the time axis. The inset shows a schematic of a population of neurons (ordinate) rank ordered by the activity peak time (abscissa). (*d*) Demonstration of a tangled representation. Multiple points along the time axis are associated with the same combination of activity levels across the three neurons causing ambiguity in the relationship between elapsed time and neural activity. (*e*) State space representation of neural activity associated with the ramping scheme shown in panel *a*. Since every neuron’s activity increases linearly, the collective activity of neurons traverses a linear trajectory. The four gray points along the trajectory represent four arbitrary neural states during the time interval. The last point (*black*) is the action initiation state. The inset shows a neural trajectory for the short condition in panel *a* (*right*). Note that this trajectory is identical to that in the long condition but evolves at a faster speed. (*f*–*h*) State space representations associated with panels *b–d*.

Any variable that increases or decreases linearly with time can be used to represent elapsed time (Figure 1a). This is the idea behind the classic clock-accumulator model in which elapsed time is represented by integration of a clock input (Treisman 1963). This so-called ramping activity has been observed throughout the brain, including the parietal cortex (Jazayeri & Shadlen 2015, Leon & Shadlen 2003, Maimon & Assad 2006), prefrontal cortex (Brody et al. 2003; Genovesio et al. 2006, 2009; Oshio et al. 2008), motor and premotor areas (Lebedev et al. 2008, Merchant et al. 2013b, Mita et al. 2009, Murakami et al. 2014, Renoult et al. 2006, Yang et al. 2024), the basal ganglia (Jin et al. 2009, Kunimatsu et al. 2018, Wang et al. 2018, Yang et al. 2024), thalamus (Matsuyama & Tanaka 2021, Tanaka 2007), cerebellum (Ashmore & Sommer 2013; Kunimatsu et al. 2018; Ohmae et al. 2013, 2017), and the entorhinal cortex (Neupane et al. 2024, Tennant et al. 2022, Tsao et al. 2022).

To produce a time interval using ramping activity, the brain must initiate the ramp at the start of the interval and generate a behavioral response when the ramp reaches a threshold for action initiation. Critically, the slope of the ramp must be such that the threshold is reached at the desired interval (see the insert in Figure 1a). How can such a system be used to produce different time intervals? Naturally, the brain must flexibly adjust either the slope of the ramp or the threshold, or both. A common observation in decision-making tasks is that the threshold is fixed (Hanes & Schall 1996, Hanks et al. 2011, Heitz & Schall 2012, Jazayeri & Shadlen 2015, Maimon & Assad 2006, Roitman & Shadlen 2002, Stine et al. 2023, Thura & Cisek 2016), suggesting that flexible timing may involve adjustments of the slope with a fixed threshold.

At first glance, it seems that the algorithm can be implemented by a neural network that integrates a tonic input whose level is flexibly adjusted. However, this solution has a counterintuitive problem. In a recurrent neural network (RNN) that integrates a tonic input, the neurons that receive the input and those that perform integration are likely to interact in ways that are not straightforward. For example, a stronger input to the integrator may cause an upward or downward baseline shift in the firing rates of neurons implementing the ramp. Moreover, an input that pushes a neuron outside of its linear operating regime may introduce nonlinearities in the ramping activity. Such deviations present a challenge for establishing an appropriate threshold; with a baseline change, the threshold should also be adjusted or the timing behavior would suffer. In sum, while the ramping scheme is intuitive and appealing, its flexible control within a neural system may involve unintuitive baseline shifts, nonlinearities, slope changes, and modulations of the threshold.

### Nonlinear Activity

The utility of nonlinear, nonmonotonic signals for representing time has been proposed in the context of chaotic neural networks (Buonomano & Laje 2010). Chaotic networks produce highly heterogeneous, nonmonotonic, and nonlinear patterns of activity (van Vreeswijk & Sompolinsky 1996). When the responses of individual neurons are highly heterogeneous, each time point becomes associated with a unique pattern of population neural activity (Buonomano & Mauk 1994, Karmarkar & Buonomano 2007, Wang et al. 2018). This one-to-one mapping, in turn, provides the means for decoding elapsed time from the population neural activity. However, certain properties of chaotic systems raise questions about their role in the brain for timing. For example, these systems can be highly sensitive to noise, are difficult to calibrate, and are even more difficult to control (Sussillo & Abbott 2009). As such, chaotic networks may not be a suitable candidate for timing in flexible timing tasks that require rapid adjustments and calibration.

Another nonlinear coding scheme represents time using concurrent oscillatory patterns of activity with different frequencies (Figure 1b) as in the striatal-beat-frequency model (Matell & Meck 2004). This model is effectively a generalization of the analog clock whose hour, minute, and second hands create three oscillators with different frequencies. We use this system to tell time by looking at the conjunctive phase of the three hands. This idea has been incorporated into models of timing involving the basal ganglia (Matell & Meck 2004, Oprisan & Buhusi 2011), but direct evidence in support of it remains wanting. Another model has proposed that time may be represented by neurons that are activated in response to external or internal events and undergo exponential decay with a diversity of time constants (Barri et al. 2022, Bright et al. 2020, Tsao et al. 2018). This scheme has certain desirable properties, including the ability to represent events concurrently across a wide range of timescales (Bright et al. 2020, Liu et al. 2019). Recordings in the cerebellum and entorhinal cortex have found signals consistent with this scheme, but their relevance to performing timing tasks and temporal judgments is not established (Barri et al. 2022, Tsao et al. 2018).

A promising alternative to generic chaotic networks is low-rank neural networks (Beiran et al. 2021, 2023; Dubreuil et al. 2022; Mastrogiuseppe & Ostojic 2018). In low-rank networks, firing rates of single neurons, despite their apparent heterogeneity, are highly coordinated. For example, the activity of every neuron may be explained as weighted sums of a small number of activity modes (DePasquale et al. 2023). In such a system, the permissible population activity patterns reside in a small subregion of the space of all possible population activity patterns (Cunningham & Yu 2014, Gallego et al. 2018, Jazayeri & Ostojic 2021, Nieh et al. 2021). This subspace is commonly referred to as the activity manifold, in reference to the concept of manifolds in mathematics that describes a well-defined and constrained subspace within a larger space.

Low-rank networks are a good candidate for timing. First, unlike unconstrained chaotic networks, they are robust to noise and perturbations (Mastrogiuseppe & Ostojic 2018, O’Shea et al. 2022) because the activity is constrained to a manifold (Beiran et al. 2023, Mante et al. 2013, O’Shea et al. 2022, Pagan et al. 2022, Pollock & Jazayeri 2020). Second, low-rank networks are relatively easy to control and are therefore particularly well-suited when flexibility is needed (Beiran et al. 2023, Egger et al. 2019, Halassa & Kastner 2017, Remington et al. 2018, Schimel et al. 2024, Sohn et al. 2019, Wang et al. 2018). Low-rank networks can be configured to use a tonic input with different strengths to adjust the speed at which activity evolves toward an action initiation threshold (Beiran et al. 2023, Remington et al. 2018, Wang et al. 2018), a solution that an idealized ramping model cannot implement.

Mounting evidence suggests that several brain systems track elapsed time using population clocks. For example, nonlinear dynamics have been observed in the dorsal medial frontal cortex (DMFC) of monkeys performing a variety of interval production tasks (Egger et al. 2019, Remington et al. 2018, Sohn et al. 2019, Wang et al. 2018). A key observation in this area is that the population activity is low-dimensional and that firing rates across neurons are highly coordinated. This observation is consistent with low-rank connectivity. It has been shown that the brain leverages this low-dimensionality to calibrate the system against inherent fluctuations and to exert control when flexibility is required. In particular, theory and modeling work inspired by neural recordings from DMFC and thalamocortical projections to DMFC have shown that both effective calibration and flexible control over the low-rank population activity in DMFC may be mediated by adjustments of a thalamocortical speed input (Wang et al. 2018, 2020). Specifically, existing neural data suggest that producing different time intervals (e.g., 1,500 versus 800 ms) is accompanied by a change in the magnitude of the thalamocortical speed input, which in turn controls the speed with which population dynamics activity in DMFC evolves over time. This speed control is evident at the level of single neurons in the form of temporal compression or stretching of firing rate modulations without altering the overall form of the nonlinear profile. In later sections, we discuss how this solution accommodates rapid calibration and flexible control through adjustments of the thalamic input.

### Sequential Activity

One variant of nonlinear coding is an ordered sequence of neuronal activations (Figure 1c). In this scheme, each neuron in the sequence reaches its peak activity at a different time, creating a chain of activations (see the inset in Figure 1c). Neurons that fire along a region of the time axis are sometimes referred to as time cells in analogy to place cells in the hippocampus (O’Keefe & Dostrovsky 1971) that fire when an animal is in a specific location. Such time cells have been reported in the rodent hippocampus (Eichenbaum 2014), in the cerebellum (Wagner et al. 2017) and cerebellum-like structures (Kennedy et al. 2014), and in the basal ganglia (Gouvêa et al. 2015, Mello et al. 2015). In this scheme, progression of the sequence corresponds to the passage of time and can thus be used to solve a variety of timing tasks.

One unique feature of the sequential scheme is that each neuron is associated with a specific point along the time axis. As such, it is particularly useful for associational learning schemes that learn the temporal relationship between events, both internal and external (Albus 1971, Eichenbaum 2014, Marr & Thach 1991). Time cells in the hippocampus may facilitate establishing the chronology of episodic memories. Time cells in the basal ganglia may organize the temporal structure of habitual behaviors and stimulus-response contingencies. Finally, time cells in the cerebellum may serve as a basis set for learning temporal events, as has been extensively studied in tasks such as overlap and trace eye-blink conditioning (Medina et al. 2000a,b). In sum, sequential coding is a special case of nonlinear coding with the unique property that it enables learning associations at the level of single neurons between time and other aspects of experience.

## A UNIFIED STATE SPACE FRAMEWORK FOR THE REPRESENTATION OF TIME

One way to visualize the coding of time is to plot the population activity pattern in a neural state space (Cunningham & Yu 2014, Ebitz & Hayden 2021, Saxena & Cunningham 2019). A neural state space is a high-dimensional Euclidean coordinate system in which each axis represents the activity of a single neuron within the population. The collective activity of all neurons in a population at a given moment is defined by a point in this state space, which is referred to as the neural state. As the activity of neurons changes over time, the neural state moves within the state space and generates a trace that is referred to as a neural trajectory (Vyas et al. 2020).

Recall that time is one-dimensional and unidirectional. These features of time bestow a necessary property of neural trajectories in order for them to represent time—they must be untangled. In other words, the neural state cannot visit the same state at different points in time or they will cause ambiguity in the temporal readout. This is in contrast to a tangled representation, which produces a trajectory that visits the same point in the neural state space at different time points (Figure 1d,h).

Each of the aforementioned coding schemes—ramping, nonlinear, and sequential—embeds untangled trajectories differently within the neural state space (Figure 1e–g). In the ramping scheme, the trajectory is a straight line that can be captured by a single dimension, with each point along the line corresponding to a specific point in time (Figure 1e). The nonlinear scheme is manifested by a more complex trajectory that reflects the ongoing modulations of firing rates as a function of time (Figure 1f). The subspace in which neural trajectories reside within the nonlinear scheme depends on the network architecture. In a chaotic network, the activity is usually high-dimensional and involves independent modulations along all axes within the state space. In low-rank models, the activity may reside within a low-dimensional manifold within the state space. The sequential scheme is a special case of the nonlinear scheme that is sparse; that is, at any point in time, only a small subset of neurons are active. This scheme causes strong rotations of the neural trajectory caused by handing off the activity from one set of axes at one point in time to another at the next point in time (Figure 1g), which increases dimensionality. Note that the estimated dimensionality across a population of neurons varies also as a result of variability, nonlinearity, and fluctuations of inputs. Therefore, in general, dimensionality is not a good proxy for distinguishing between coding schemes.

Despite the differences across coding schemes, conceptualizing the population activity as a trajectory through neural state space can be highly instructive. First, all relevant schemes must produce untangled trajectories—each position on the neural trajectory has a one-to-one mapping to a point in the time axis. Second, irrespective of the coding scheme, the neural state must correspond to the subjective estimate of elapsed time. For example, faster movement along the trajectory would lead to an earlier behavioral response. Third, the geometry of the neural trajectory could inform the nature of behavioral variability. For example, if elapsed time is decoded by projecting activity onto a predetermined dimension, deviations orthogonal to this dimension would not impact timing behavior. In contrast, noise that is aligned with this dimension would have a direct impact on timing behavior (Meirhaeghe et al. 2021, Moreno-Bote et al. 2014, Sohn et al. 2019, Wang et al. 2020). Finally, the structure of the neural activity in the state space is constrained by the coupling between neurons. As such, understanding this structure can provide valuable information about the underlying neural circuits that may not be apparent from the activity of single neurons (Vyas et al. 2020). Notably, these insights derived from the state space representation are common to all coding schemes even though they correspond to different phenomenologies. For example, adjustments of speed would manifest as a change in the slope of the ramp in the ramping scheme, as a change of temporal scaling (compression versus stretching) in nonlinear schemes, and as a change in the progression of the sequence in the sequential scheme.

As we discuss the virtues of the state space framework, it is important to emphasize some caveats. Neural state space analyses often treat neurons the same irrespective of anatomy. This approach warrants caution. Specifically, when the neurons in a population are composed of distinct subpopulations with different properties and projections, one must take those distinctions into account to arrive at valid explanations of the underlying biology (Churchland & Lisberger 2005, Fee et al. 2004, Hirokawa et al. 2019, Komiyama et al. 2010, Movshon & Newsome 1996, Sommer & Wurtz 2008, Steinemann et al. 2023, Xiong et al. 2015, Xu et al. 2022). Therefore, the organization of neural trajectories in the state space is best thought of as a constraint in a virtuous cycle of discovery composed of (*a*) analysis of neural activity using increasingly more precise measurements of both physiology and anatomy, (*b*) synthesis using increasingly more accurate models, and (*c*) causal validation of mechanisms using perturbations of task contingencies and neural activity.

## NEURAL DYNAMICS THROUGH THE LENS OF TIMING

The dynamical motifs associated with encoding time are not specific to timing and have been observed in diverse behavioral tasks. Ramping activity plays a crucial role in motor planning and decision making (Gold & Shadlen 2007, Schall 1995). Nonlinear activity is a common observation in frontal and prefrontal cortex in animals performing working memory tasks (Rajalingham et al. 2022, Tang et al. 2020, Xie et al. 2022). Sequential activity is widespread in perceptual, cognitive, and motor tasks (Crowe et al. 2010, Fujisawa et al. 2008, Hahnloser et al. 2002, Harvey et al. 2012, Ikegaya et al. 2004, Jin et al. 2009, Koay et al. 2022, Nádasdy et al. 1999, Seidemann et al. 1996). Therefore, understanding the neural basis of these dynamical motifs in simple timing tasks can have profound implications for a general understanding of the linkage between neural dynamics and behavior. Here, we focus on a body of recent work within the context of various time interval production tasks that have informed broad questions about how cortical dynamics can be flexibly controlled and rapidly calibrated via thalamic inputs (Wang et al. 2018, 2020). We then conclude with a hypothesis regarding general principles of flexible control and rapid adaptation of cortical dynamics through the thalamus and highlight open questions about the roles of ascending pathways originating in the cerebellum and basal ganglia in supplying the thalamic signals.

## FLEXIBLE CONTROL OF NEURAL DYNAMICS

Consider a variant of the interval production task termed the cue-set-go (CSG) task (Wang et al. 2018). In this task, monkeys are presented with a visual cue at the fixation point whose color instructs them to produce either a short (red for 800 ms) or long (blue for 1,500 ms) interval after the presentation of a set stimulus. Critically, the two intervals are interleaved on a trial-by-trial basis, requiring that the animals quickly and flexibly adjust their timing behavior based on the cue.

**Figure 2:**
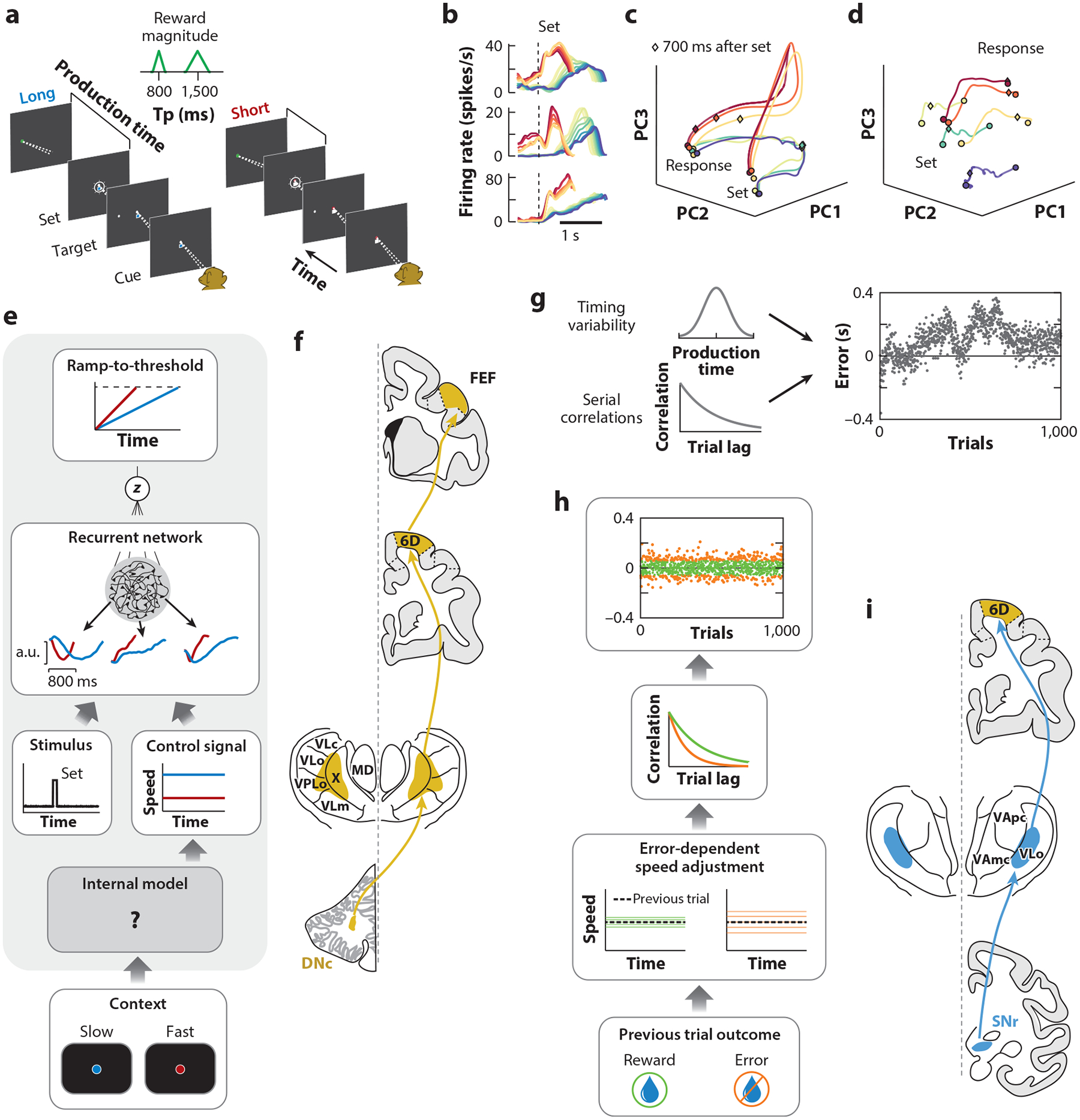
Control and calibration of neural dynamics for flexible timing behavior. (*a*) Schematic of a simple time interval production task in monkeys. The animal fixates a central spot (cue) whose color serves as a cue for whether to produce a short 800-ms (*red*) or long 1,500-ms (*blue*) interval. After the presentation of a saccadic target (target), a flash (set) initiates the time interval. The animal has to proactively make a saccade to the target at the desired time. The reward magnitude scales with accuracy (*green triangular function*). (*b*) Firing rate as a function of time for three example neurons in DMFC sorted based on the produced time interval with shades of red for the short time interval and shades of blue for the long time interval. Evidently, the response profile for each neuron remains largely unchanged except for an overall temporal scaling (compression for shorter intervals and stretching for longer ones). (*c*) Neural trajectories associated with a population of DMFC neurons plotted within the subspace spanned by the first three principal components of the full state space. Neural trajectories are largely matching within the first two principal components. (*d*) Neural trajectories, similar to those shown in panel *c*, for the thalamus. Thalamic responses are relatively stable throughout the trial and vary in magnitude for different time intervals. (*e*) Schematic depicting speed control of dynamics through contextual inputs. An internal model, the form of which is unknown, learns to modulate the magnitude of a tonic control signal in accordance with the context cue. The control signal sets the speed of nonlinear, nonmonotonic activity within a recurrent network in response to the set stimulus. A weighted sum of units within the recurrent network produces ramping activity, the slope of which depends on the speed of dynamics in the recurrent network. When this ramp exceeds a threshold, the action is initiated. (*f*) A cerebello-thalamocortical circuit hypothesized to be involved in the adjacent steps in panel *e*. Schematics depict coronal sections of the rhesus macaque brain. The cerebellum, thought to be involved in forming internal models, projects to the ventral lateral thalamus via the DNc. Thalamus is thought to provide a control signal to area 6D of frontal cortex (DMFC). Area 6D provides input to the FEF, which displays ramping activity in oculomotor tasks. (*g*) Internal sources of variability for timing behavior (*left*). Without calibration, correlated noise across trials produces slow drifts in timing behavior, resulting in large errors (*right*). (*h*) Calibration of timing behavior through error-dependent adjustment of speed. Increased variability in the control signal following an error reduces serial correlations, facilitating calibration. (*i*) A basal ganglia–thalamocortical circuit thought to be involved in reward-based calibration of neural dynamics. Abbreviations: DNc, dentate nucleus; DMFC, dorsal medial frontal cortex; FEF, frontal eye field; SNr, substantia nigra pars reticulata; VA_mc_, ventral anterior nucleus, magnocellular division; VA_pc_, ventral anterior nucleus, parvocellular division; VL_c_, ventral lateral caudal nucleus; VL_m_ ventral lateral medial nucleus; VL_o_, ventral lateral oral nucleus; VPL_o_, ventral posterior lateral oral nucleus. Panels *a–d* adapted from Wang at al. (2018) (CC BY 4.0).

Neurons in the DMFC recorded during the CSG task display nonlinear, nonmonotonic activity that is temporally compressed on short interval trials and stretched on long interval trials (Figure 2b), a phenomenon that has been termed temporal scaling (Wang et al. 2018). When all the neurons are temporally scaled by a certain factor without changing the response profile, the population activity goes through the same continuum of states, but only at a different speed. Therefore, in the state space, temporal scaling manifests as similar neural trajectories that evolve at different speeds (faster for shorter intervals; Figure 2c).

To trace the source of temporal scaling, Wang et al. (2018) recorded from thalamic neurons projecting to DMFC. Remarkably, in the thalamus, unlike DMFC, the main activity mode was not temporal scaling. Instead, neurons exhibited tonic activity whose magnitude was adjusted by the visual cue (Figure 2d). In other words, the thalamic neurons provided a context input. This observation created a puzzle. How could tonic changes in the thalamic input control the speed of dynamics in DMFC? To gain insight into this property, it is useful to think of temporal scaling as a change in an effective time constant. For an exponential decay, for example, a change in time constant leads to temporal scaling. By analogy, the contextual input in the CSG task seems to change the effective time constant of the system. How can a tonic input to a dynamical system change time constants?

To tackle this question, let us start by assuming that DMFC acts as a linear dynamical system, with an instantaneous state, denoted as *X*. As a dynamical system, the change in the state, d*X*/d*t*, is determined by a linear transformation of the state, *MX*, plus an external input, *I*_ext_. These assumptions can be distilled into the canonical equation of a linear dynamical system:

$$\tau \frac{\text{d}X}{\text{d}t}=MX+I_{\text{ext}}.$$

Is it possible to change the time constant of this system by changing the input, say, by applying a gain factor, *g*? We can assess this straightforwardly by adjusting the input and some rearrangement:

$$\tau \frac{\text{d}X}{\text{d}t}=MX+gI_{\text{ext}},$$

$$\tau /g\frac{\text{d}X}{\text{d}t}=(M/g)X+I_{\text{ext}}.$$

Comparing this equation to the original one, the time constant is divided by the gain factor, but the linear transformation, denoted by *M*, is also divided by the same gain factor. In other words, a change to the time constant requires a system with a different effective connectivity pattern. This observation applies broadly to linear dynamical systems: A change in input cannot change the time constant without changing other aspects of the dynamics. How then does the system harness the change in input for speed adjustment? One possibility is that the input is neuromodulatory. Neuromodulatory signals can indeed change synaptic connections and may therefore be able to alter effective connectivity in a way that mimics a change in time constant (Lee & Dan 2012).

This solution was proposed in a recent theoretical study that sought to explain how the motor cortex might generate movements at different speeds (Stroud et al. 2018). The same solution may also apply in the context of the CSG task. However, thalamocortical projections are not directly associated with the brain’s neuromodulatory systems. Can a nonmodulatory input cause an effective change in connectivity? To address this question, Wang et al. (2018) trained RNN models consisting of simple nonlinear units to use a tonic, thalamic-like input to perform the CSG task. Once trained, units within these networks recapitulate the heterogeneous, nonlinear, and nonmonotonic activity of single neurons observed in DMFC as well as temporal scaling across different intervals. Importantly, temporal scaling was evident even though the model did not include any neuromodulatory components. Reverse engineering the trained RNNs offered an alternative solution for speed control. Stronger input pushed neurons toward the saturating nonlinearity of their input-output response curve. When neurons function near their saturating nonlinearity, their response becomes less sensitive to ongoing dynamics. It is easy to appreciate this by considering the extreme case of a neuron that is fully saturated. Such a neuron will be completely unresponsive to dynamics. In sum, the RNNs were able to convert the input to a speed control signal by exploiting the nonlinearities in the system.

The finding that speed control may be mediated through nonlinearities bears on our initial discussions of various coding schemes for timing. We presented linear (ramping) and nonlinear coding schemes as alternatives for coding time. However, the combination of physiology and modeling in CSG suggests that flexible control may impose important constraints on which coding scheme a neural system might adopt. Specifically, as exemplified by activity patterns in DMFC during the CSG task, when a neural system is required to produce different time intervals flexibly, it may follow that contextual cues alter the neural state such that the nonlinearities enable the input to act as an effective knob for speed control (Figure 2e). Since the original CSG study, several other experimental studies have substantiated this finding (Betancourt et al. 2023, Egger et al. 2019, Meirhaeghe et al. 2021, Remington et al. 2018, Sohn et al. 2019). This idea has also been validated and developed in a theoretical study showing that low-rank nonlinear neural networks augmented with tonic control inputs can generalize their timing functions (Beiran et al. 2023). One point of interest in these modeling studies is that RNN units emulate the observed neural data in DMFC when the action initiation in the model is established through threshold crossing mechanisms. This observation suggests that thresholding operations may be a key constraint on systems that use temporal scaling and speed control for flexible timing behavior.

The results above suggest that the contextual cue in the CSG task leads to a change in tonic thalamic activity, which changes the speed of dynamics in DMFC and the slope of ramp-to-threshold dynamics in downstream regions involved in enacting the motor plan (Figure 2e). This begs the question of how exactly the transformation occurs between the color of the cue and the thalamic signal. A powerful idea is that the brain leverages internal models to control such signals. Internal models have been studied extensively in the context of motor control, in which predictions about the sensory consequences of actions enable precise and adaptable movement. Decades of research suggest that the cerebellum plays a critical role in implementing internal models for motor control. An intriguing hypothesis is that similar internal models are used to predict how modulations to internal signals, such as thalamic activity, will impact cortical activity, enabling precise control of cortical dynamics (Figure 2f). Indeed, the ventral lateral thalamus receives direct input from the cerebellar dentate nucleus (Strick et al. 2009), which is thought to be important for nonmotor functions of the cerebellum. Whether and how the cerebellum is involved in the control of cortical dynamics, and how it interacts with regions of the brain that represent abstract rules and contexts, will be important avenues for future work. The mechanism of speed control described above lays the foundation for exploring this question.

## CALIBRATION OF NEURAL DYNAMICS

The nervous system is highly stochastic. Neural activity patterns across the brain exhibit high degrees of variability. This variability has been documented at multiple spatial and temporal scales, in many brain areas, and during a wide range of behavioral tasks and states (Churchland et al. 2010, Dhawale et al. 2017, Goris et al. 2014, Renart & Machens 2014, Shadlen & Newsome 1998). Plasticity also causes structural changes at relatively slower timescales, causing additional variability (Mongillo et al. 2017, Rule et al. 2019). In the absence of an external reference and/or feedback, the brain has no means to detect this variability. Critically, variability that is left unchecked can lead to large cumulative errors. Therefore, the brain must continually monitor external observations and compare them to predictions and expectations to remain calibrated. Not all types of variability can be countered. For example, when trial-by-trial errors are independent, attempting to correct them is futile and does nothing more than chase noise with no improvement in outcome. The scenarios in which calibration is warranted are those that involve structured errors. Therefore, the process of calibration is rather involved; the brain must monitor errors, tease apart scenarios where errors are structured, and make adjustments accordingly. This process is a fundamental aspect of brain function, but its underlying mechanisms are not known.

In a follow-up study, Wang et al. (2020) tackled this question by studying timing errors in the CSG task. On each trial, the subjects (both humans and monkeys) received positive feedback on small-error trials and negative feedback on large-error trials. This feedback helped the subject determine when an error occurred but provided no information about the sign of error, that is, whether the produced interval was too short or too long. Analysis of errors across trials revealed multiple timescales of variability (left side of Figure 2g). Some errors were unstructured across trials and thus could not be effectively countered. Others featured serial correlations. Correlated errors, if left unchecked, would accumulate indefinitely, causing large drifts in subjects’ production times over the course of a session (right side of Figure 2g). However, production times remained well-matched to the target interval, indicating that a calibration process is at play. Analysis of behavioral variability following positive and negative feedback trials revealed a rational algorithm for calibration. Positive feedback was followed by reduced variability and increased serial correlations promoting repetition in behavior. In contrast, negative feedback led to increases in variability and reductions in serial correlations promoting explorations away from the previous incorrect behavior (Figure 2h). These findings highlight an ongoing calibration process that leverages variability to control the degree of exploration versus exploitation depending on prior feedback.

Recordings from multiple brain areas, including DMFC, the caudate nucleus, and DMFC-projecting thalamus, indicated that thalamic signals may play a particularly important role in this process (Wang et al. 2020). The thalamic responses that best predicted the serial correlations in production intervals increased and decreased their variability in accordance with feedback in the preceding trial. If thalamus is indeed controlling the speed of dynamics in DMFC with a one-dimensional input, then such alignment would be necessary for recalibration. This alignment was not observed in DMFC or the caudate nucleus. What is the origin of reward-dependent adjustment in the variability of thalamic activity? Strong evidence points to the output structures of the basal ganglia—the substantia nigra and the globus pallidus—which supply input to the ventral lateral thalamus and are critical for reward-dependent behavior (Goldberg et al. 2013) (Figure 2i). Indeed, in the song bird, it is well-appreciated that injection of variability into the motor system by the basal ganglia is an important component for learning and adaptation of song production (Dhawale et al. 2017, Garst-Orozco et al. 2014, Ölveczky et al. 2005).

Often, immediate reinforcement is unavailable. Even in these cases, timing behavior remains relatively well-calibrated. For example, musicians can maintain a tempo over long timescales without the use of a metronome. Little is known about how calibration is achieved without explicit feedback, but internal models that learn the temporal structure of the environment are likely critical. Such models make predictions about the timing of events. Systematic errors in these predictions due to slow drift can, in theory, be used for recalibration. Unlike reinforcement, prediction errors resulting from internal models are signed—they inform the direction in which correction should occur—making them particularly useful for calibration (Izawa & Shadmehr 2011). Indeed, recordings from DMFC in monkeys performing a temporal integration task show that internal models are used to adjust speed inputs based on temporal predictions, improving timing behavior (Egger et al. 2019). Strong evidence suggests that the cerebellum is a key neural substrate for these internal models (Wolpert et al. 1998). Recordings from a variety of timing tasks across different species suggest that the cerebellum predicts the timing of events in the environment (Garcia-Garcia et al. 2024; Johansson et al. 2016; Medina et al. 2000b; Ohmae et al. 2013, 2017; Okada et al. 2022; O’Reilly et al. 2008; Wagner et al. 2017). Furthermore, cerebellar insult in humans causes deficits in timing (Ivry & Keele 1989, Ivry et al. 2002). How reward-based calibration interacts with calibration through internal models, and whether they have overlapping neural substrates, will be an important avenue for future work.

## A GENERAL PRINCIPLE OF PARAMETRIC CONTROL OF CORTICAL DYNAMICS

**Figure 3:**
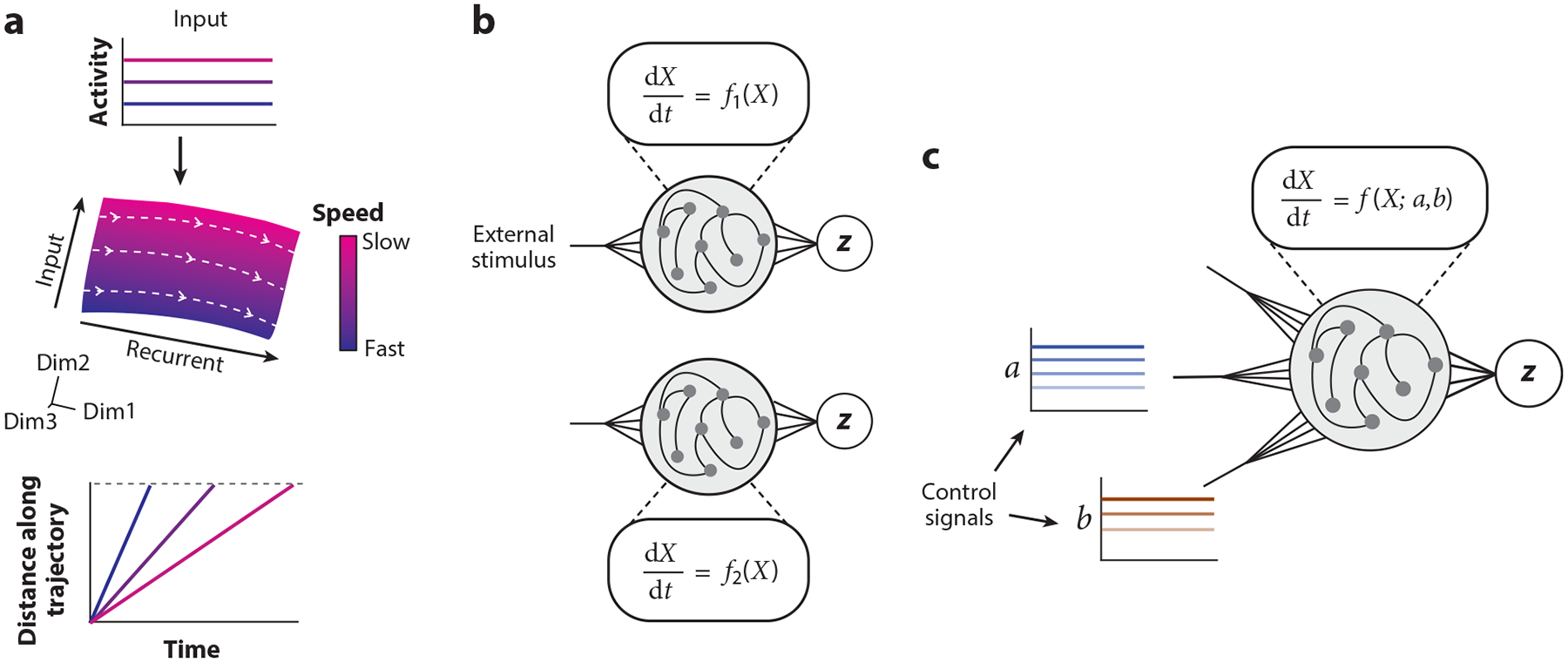
A general principle of parametric control of neural dynamics. (*a*) Parametric control of speed through tonic thalamic input. (*Top*) Tonic input to a recurrent network conveys the desired speed of neural dynamics. (*Middle*) Recurrent connections give rise to a manifold on which trajectories are invariant along the recurrent direction but vary in their speed depending on the strength of the input. (*Bottom*) Distance as a function of time for three example trajectories that vary along the input dimension. (*b*) Lack of parametric control in standard recurrent neural networks. In such networks, changes to synaptic weights are required to produce two different dynamical regimes, *f*1 and *f*2, in response to the same stimulus. (*c*) Parameterization of dynamics through recurrent connections. The recurrent connections of the network specify the parameterization of the dynamics [*f* (*X*; *a*,*b*)], which, through experience or development, becomes well-matched to the statistical structure of the environment. The dimensions of the dynamics that need to be quickly controlled and calibrated (*a* and *b*) are factored out as inputs, serving as control signals that specify the appropriate parameters.

The results described above evoke an architecture in which the speed of complex dynamics in cortex is parametrically controlled by relatively simple, tonic inputs from the thalamus (Figure 3a). This architecture is appealing for a variety of reasons. It enables flexible control of speed of cortical dynamics without the need to adjust connectivity in cortical circuits. It also offers a locus for calibrating the speed of neural dynamics in the presence of internal and external fluctuations. If cortical activity is analogous to music being played through a speaker, then thalamic activity is analogous to a knob that can be adjusted and calibrated to control the tempo without impacting the music. A natural question is how this knob in the thalamus gets set. The area of thalamus where this control signal was found is a likely target of output nuclei in the cerebellum and basal ganglia (Figure 2f,i). Therefore, we speculate that these subcortical areas are crucially involved in the control setting of this knob.

Parametric control of speed is possible because the recurrent connections among neurons in DMFC manifest an activity manifold that is well-matched to flexible timing tasks. Specifically, trajectories along the manifold are largely invariant to translations along the input dimension, varying primarily in their speed (Figure 3a). Such invariance is the key feature that enables flexible control, calibration, and generalization to novel conditions through simple adjustments to the thalamic input, and it arises naturally in low-rank networks trained on flexible timing tasks (Beiran et al. 2023). This is in contrast to chaotic networks that adjust their dynamics through changes in synaptic weight (Sussillo & Abbott 2009). In such networks, a change in the input can place the network in an entirely different dynamical regime, leading to activity patterns that are unpredictable and difficult to control. Moreover, achieving a specific change in the dynamics requires retraining the network’s synaptic weights (Figure 3b), potentially causing catastrophic forgetting of previously learned conditions.

We propose a general principle of flexible and controllable cortical dynamics (Figure 3c). Namely, the abstract structure of the environment or task is encoded implicitly in the structure of recurrent connections between neurons. These connections shape manifolds that parameterize the dynamics such that they are well-matched to the task at hand. The parameters that ought to be flexible are factored out as control inputs. Within this functional architecture, flexibility is achieved through modulations of these inputs, facilitating generalization. The work we reviewed in the context of interval timing is an instance of this proposal: By virtue of factorizing the speed signal to the thalamus, the system enables parametric control over the speed of cortical dynamics.

Flexibility and generalization are hallmarks of human cognition. If insights from the neurobiology of flexible timing were to apply more broadly, we would predict that the cognitive architecture of the human brain has three key functional building blocks: (*a*) manifolds that suitably capture abstract properties of our environment, (*b*) control signals that furnish parametric control over the dynamics within those manifolds, and (*c*) neural substrates for learning such control. An intriguing idea is that these three building blocks are in part instantiated by the evolutionary coexpansion of the prefrontal cortex to establish manifolds, thalamus for supplying control, and lateral cerebellum for adaptive learning of the control. In this view, a key function of the ascending cerebellar pathways is to exert parametric control over cortical dynamics (Gao et al. 2018).

Speed adjustment may be just one of many dimensions of cortical control mediated by simple inputs. Control of visual attention may be another (Halassa & Kastner 2017). The structured connectivity of neurons in the visual cortex creates a fixed spatial map of the visual environment. Attended locations on this map, however, are dynamic and can be adjusted on demand. It is thought that attentional control signals are factorized via spatial priority maps instantiated by other brain areas that provide direct or indirect input to visual cortex (Andersen & Buneo 2002, Armstrong et al. 2009, Krauzlis et al. 2013, Moore & Armstrong 2003, Moore et al. 2003). These inputs may be routed through the thalamus (McAlonan et al. 2006, 2008; Schmitt et al. 2017) and can ameliorate visual processing by increasing signal and reducing noise in targeted visual cortical neurons (Briggs et al. 2013, Maunsell 2015) and by altering functional connectivity between visual areas (Ruff & Cohen 2016, 2019; Srinath et al. 2021). Recent work has shown that such changes can emerge from simple, spatially selective inputs due to circuits that implement normalization (Lee & Maunsell 2009, Ni & Maunsell 2019, Ni et al. 2012, Ruff & Cohen 2017, Verhoef & Maunsell 2017). Given what we have learned about the control of speed through simple tonic inputs, we speculate that one reason why such normalization circuits are ubiquitous in visual cortex is because they render complex attentional effects controllable by simple inputs. Identification of shared principles and architectural constraints such as those in speed and attentional control would constitute a major advance in our understanding of flexible cognitive behavior.

## CONCLUSIONS AND OPEN QUESTIONS

A major goal of neuroscience is to identify principles that govern how the brain represents latent variables and how these representations support flexible behavior. Here, we have argued that this endeavor is simplified in the context of timing behaviors because of time’s unique properties among the senses: (*a*) it is one-dimensional, (*b*) it is unidirectional, and (*c*) our sense of time relies on internally generated dynamics rather than external stimuli. These properties have enabled deep insights into how time is represented by populations of neurons by providing constraints on how activity patterns relate to the latent variable of interest. One critical insight is that the brain represents time using a variety of different schemes. Analysis of single neurons suggests that these schemes differ drastically, whereas analysis of the neural state space highlights their similarities. Specifically, the state-space framework reveals that commonly observed representations of time produce smooth, untangled neural trajectories, such that each point in time is associated with a unique pattern of activity. Despite these commonalities, different schemes are likely to facilitate different functions. For example, ramping activity may be particularly well-suited to interval production and anticipation, whereas sequential activity is well-suited for learning the temporal relationships between disparate events.

Across the schemes, the state-space framework also reveals that the speed of neural trajectories is closely related to subjective perception of time and interval production. We highlighted recent work that showed that control of this speed underlies flexible timing behavior. Such adjustment of behavior is too fast to rely on changes to synaptic weights. Instead, we explored how a tonic input signal, likely originating from the thalamus, can adjust the speed of dynamics in cortex through interactions with nonlinearities and appropriately structured manifolds that manifest from low-rank recurrent connectivity. We also explored how the same speed control mechanism can be used to combat variability inherent to the brain through error-based calibration of the tonic thalamic signal. The insights into the control and calibration of dynamics in cortex through tonic inputs in the context of flexible timing led us to speculate about a more general principle in the brain. Namely, recurrent connections in cortex implicitly learn the structure of the environment, forming manifolds that enable parametric control of dynamics through simple, low-dimensional inputs.

The work described above raises a number of open questions. Below we highlight a few that we believe hold particular promise for revealing neurobiological principles of neural coding and cognitive control:

- How do putative control signals from the thalamus interact with cortical recurrent activity?
- What brain regions control thalamic input, and how do they learn to do so?
- How are multidimensional control signals mediated by the thalamocortical pathway?

These questions are rendered tractable by the simplifying properties of time and the insights described in this review. Their answers are well-posed to reveal fundamental principles of brain function more generally.
